# Reviewer acknowledgement 2013

**DOI:** 10.1186/1758-5996-6-9

**Published:** 2014-01-29

**Authors:** Daniel Giannella-Neto, Marilia de Brito Gomes

**Affiliations:** 1 Universidade Nove de Julho (UNINOVE), São Paulo, Brazil; 2 Universidade Estadual do Rio de Janeiro, Rio de Janeiro, Brazil

## Abstract

**Contributing reviewers:**

The Editors of *Diabetology and Metabolic Syndrome* would like to thank all our reviewers who have contributed to the journal in Volume 5 (2013).

## 

Rohana Abdul Ghani

Malaysia

Nader Abraham

United States of America

Kerstin Abshagen

Germany

Allen Adolphe

United States of America

Aila Ahola

Finland

Carlos Aita

Brazil

Akinyele Akinlade

Nigeria

Francesco Andreozzi

Italy

Michael Andrew

United States of America

Toby Andrew

United Kingdom

Giovanni Annuzzi

Italy

Keiko Arai

Japan

Adria Arboix

Spain

Eloisa Arbustini

Italy

Yoshimasa Aso

Japan

Vasilios Athyros

Greece

Anna Aulinas

Spain

Mirela Azevedo

Brazil

Akira Babazono

Japan

Usman Baber

United States of America

Rosa Bacchetta

Italy

Wish Banhiran

Thailand

Maja Baretic

Croatia

Emil Daniel Bartels

Denmark

Nicolas Bazan

United States of America

Nina Berentzen

Netherlands

Franco Berrino

Italy

Darlene Berryman

United States of America

Julie Bienertova-Vasku

Czech Republic

Andreia Biolo

Brazil

Vincent Blasco-Baque

France

Pascal Bousquet

France

Phillip J Brantley

United States of America

Carrie Breton

United States of America

Anna Brini

Italy

Richard Brunner

Austria

Christa Buechler

Germany

Doina Bunescu

Romania

Emilee Burgess

United States of America

Claudio Cabello-Verrugio

Chile

Weibin Cai

China

Cleber Camacho

Brazil

Franco Cavalot

Italy

Selim Cellek

United Kingdom

Gwo-Shing Chen

Taiwan

Robert Chilton

United States of America

Michel Chretien

Canada

Roberta Cobas

Brazil

Daniel Cobaugh

United States of America

Ricardo Cohen

Brazil

Shane Coleman

United States of America

Eva Conceicao

Portugal

Maria Lucia Correa-Giannella

Brazil

Carlos Couri

Brazil

Ana B Crujeiras

Spain

Cecilia Dall'Aglio

Italy

Zari Dastani

Canada

Erick de Oliveira

Brazil

Celia de Souza Batista

United States of America

Miguel Debono

United Kingdom

Petronella E Deetman

Netherlands

Sylvie Dejager

France

Buket Demirci

Turkey

Anne Drougard

France

Ken Ebihara

Japan

Brendan Egan

Ireland

Garry Egger

Australia

Cihangir Erem

Turkey

Ahmet Goktug Ertem

Turkey

Radina Eshtiaghi

Iran

Jeffery Evans

United States of America

Igor Fernandes

Brazil

Devaka Fernando

United Kingdom

Sandra Roberta Gouvea Ferreira

Brazil

Juraj Fillo

Slovakia

Laura Fonken

United States of America

Laercio Franco

Brazil

Eliete Dalla Corte Frantz

Brazil

Jeffrey Freeman

United States of America

Atsunori Fukuhara

Japan

Trudy Gaillard

United States of America

Baptist Gallwitz

Germany

Giorgio Gandaglia

Italy

Amador Garcia

Spain

M K Garg

India

Rebecca Geddes

United Kingdom

Lia Gentil

Canada

Melvin George

India

Neoklis Georgopoulos

Greece

Tamara Beres Lederer Goldberg

Brazil

Patricia Gomes

Brazil

Rodrigo Gomes

Brazil

Rosita Gomes

Brazil

Brett Gordon

Australia

Maria Gottlieb

Brazil

Dimitrios Goulis

Greece

Bianca Gregorio

Brazil

Giuseppe Grosso

Italy

Ashok Grover

Canada

Orlando Gutierrez

United States of America

Ali Hajeer

Saudi Arabia

Alfredo Halpern

Brazil

Katrine Bagge Hansen

Denmark

Michio Hashimoto

Japan

Takeshi Hashimoto

Japan

Karim Hmadcha

Spain

Leanne Hodson

United Kingdom

Naomi Holman

United Kingdom

Tim Holt

United Kingdom

Yoshiyuki Horio

Japan

Frank Howarth

United Arab Emirates

Yinan Hua

United States of America

Ernesto Iadanza

Italy

Viorica Ionut

United States of America

Shinichi Ishii

Japan

Ellis Jensen

United States of America

Majken Jensen

United States of America

Bo Ji

United States of America

Rocio Jimenez Galan

Spain

Tianru Jin

Canada

Kohei Kaku

Japan

Zdravko Kamenov

Bulgaria

Katerina Kankova

Czech Republic

Kaffer Kara

Germany

Dimitrios Karaiskos

Greece

Newton Kara-Junior

Brazil

Chandan Karmakar

Australia

Niki Katsiki

Greece

Gurpreet Kaur

Malaysia

Harmeet Kaur

United States of America

Aaron Kelly

United States of America

Romesh Khardori

United States of America

Kaivan Khavandi

United Kingdom

KyoungKon Kim

Korea South

Mary Korytkowski

United States of America

Martin Krssak

Austria

Setor Kunutsor

United Kingdom

Thomas Lagace

Canada

Sophie Lalande

Canada

Lena Lämmle

Germany

Rodrigo Lamounier

Brazil

Julia Lamparter

Germany

Annunziata Lapolla

Italy

Srinivas Laxminarayan

United States of America

Jimmy Lee

Singapore

Ming-Che Lee

Taiwan

Silmara Leite

Brazil

Meenakshi Lella

India

Dongmei Li

United States of America

Charalampos Liakos

Greece

Fabio Lima

Brazil

Lisa Lincz

Australia

Fabio Lira

Brazil

Shuainan Liu

China

Tobias Lohmann

Germany

Heno Lopes

Brazil

Ru-Band Lu

Taiwan

Bing Luan

United States of America

Claudia Luevano-Contreras

United States of America

Michael Lustgarten

United States of America

Fang Ma

China

Clare Mackie

United Kingdom

Shiro Maeda

Japan

Prokopios Magiatis

Greece

Costan Magnussen

Australia

Daniela Magro

Brazil

Maria M Malagon

Spain

Steven Malin

United States of America

Robin Maskey

Nepal

Ben Maskrey

United Kingdom

Kawaljit Matharoo

India

Alessandra Matheus

Brazil

Morihiro Matsuda

Japan

Didac Mauricio

Spain

Janice Mayne

Canada

Maija Mednieks

United States of America

Karla Melo

Brazil

Vadayath Usha Menon

India

Kotb Metwalley

Egypt

Pieter Meyns

Belgium

Ilias Migdalis

Greece

Marian Mokan

Slovakia

Babak Mokhlesi

United States of America

Myeong Hee Moon

Korea South

Rodrigo Moreira

Brazil

Ryuichi Morishita

Japan

Yusuke Moritoh

Japan

Bernhard Moser

Austria

Kristin Mühlenbruch

Germany

Giuseppe Musumeci

Italy

Ramanathan Muthiah

India

Gabriel Mutungi

United Kingdom

Tsutomu Nakahara

Japan

Louise Naylor

Australia

Carlos Negrato

Brazil

Christopher Newton

United States of America

Orla Neylon

Australia

Nik Ruzyanei Nik Jaafar

Malaysia

Budimka Novakovic

Serbia

Luciana Nucci

Brazil

Alistair Nunn

United Kingdom

Thomas Nyström

Sweden

Eiji Oda

Japan

Anthonia Ogbera

Nigeria

Jee-Young Oh

Korea South

Koji Ohashi

Japan

Altan Onat

Turkey

Nada Orsolic

Croatia

Rytas Ostrauskas

Lithuania

Noriyuki Ouchi

Japan

Vilborg Palsdottir

Sweden

Giuseppe Papa

Italy

Dimitrios Papamargaritis

United Kingdom

Nikolaos Papanas

Greece

Candida Parisi

Brazil

Chan Kee Park

Korea South

Marisa Passarelli

Brazil

Michael Lynge Pedersen

Greenland

Juan Pedro-Botet

Spain

Dee Pei

Taiwan

David Pham

United States of America

Jean-Christophe Philips

Belgium

Meroni Pier Luigi

Italy

Gustavo Duarte Pimentel

Brazil

Alexander Pisarchik

Spain

Maria Ponto

United Kingdom

Rosalinde Poortvliet

Netherlands

Sergio Portal-Núñez

Spain

Jonato Prestes

Brazil

Luca Puccetti

Italy

William Quick

United States of America

Rajendra Raghow

United States of America

Rishi Rattan

United States of America

Flavio Reis

Portugal

Suman Rice

United Kingdom

Antonello Rigamonti

Italy

Sherine Rizk

Egypt

Christian Roberts

United States of America

David Rodbard

United States of America

Leonardo Roever

Brazil

Marilza Rudge

Brazil

Ranabir Salam

India

Joao Eduardo Salles

Brazil

Stefanie Samietz

Germany

Beatriz Schaan

Brazil

Janice Schwartz

United States of America

Peter Schwarz

Germany

Heike Serke

Germany

Jose Silva

Brazil

Marta Silva

Portugal

Loreana Silveira

Brazil

Borkoski Barreiro Silvia Andrea

Spain

David R Sinacore

United States of America

Balwinder Singh

United States of America

Giorgia Sisino

Italy

Ake Sjoholm

Sweden

Karolina Skibicka

Sweden

Min-Woong Sohn

United States of America

James Sowers

United States of America

Eberhar Spanuth

Germany

J David Spence

Canada

Bessie E Spiliotis

Greece

Norbert Stefan

Germany

Manuela Stefanelli

Italy

Bernd Stegmayr

Sweden

Jian-bin Su

China

Milton Suarez-Ortegón

Colombia

Joanna Suliburska

Poland

Grazyna Sypniewska

Poland

Yohei Takai

Japan

Xiao Tan

Finland

Giovanni Tarantino

Italy

Alexander Tenenbaum

Israel

Kamani Tennekoon

Sri Lanka

Hemant Thacker

India

Neha Thakur

India

Ranjeny Thomas

Australia

Rebecca Thomson

Australia

Hao-ming Tian

China

Irén Tiberg

Sweden

Maryam Tohidi

Iran

Inês Tomada

Portugal

Avinash Tope

United States of America

Apostolos Tsapas

Greece

Maria Teresa Tusie-Luna

Mexico

Konstantinos Tziomalos

Greece

Raivo Uibo

Estonia

Melanie van der Klauw

Netherlands

Sergio Vencio

Brazil

Michel Vernay

France

David Vesely

United States of America

Felipe Vilella Mitjana

Spain

Algis Vingrys

Australia

Giovanni Viscogliosi

Italy

Giovanni Vitale

Italy

Charles Wade

United States of America

Hayfaa Wahabi

Sudan

Bernardo Leo Wajchenberg

United Kingdom

Kenji Wakai

Japan

Gareth Wallis

United Kingdom

Chih-Yuan Wang

Taiwan

Yaoming Wang

United States of America

Yi-Xin (Jim) Wang

China

Li-Na Wei

United States of America

Martin O Weickert

United Kingdom

Stephen Weinrib

United States of America

Debra Weinstein

United States of America

Karen Whalen

United States of America

Alistair Williams

United Kingdom

Michael Wolfgang

United States of America

Jeong-taek Woo

Korea South

Yen-Wen Wu

Taiwan

Edward Wylegala

Poland

Tamaki Yamada

Japan

Sho-ichi Yamagishi

Japan

Kennosuke Yamashita

Japan

Toshimasa Yamauchi

Japan

Li Yang

United States of America

Wei-Shiung Yang

Taiwan

Yen Kuang Yang

Taiwan

Zhihong Yang

Switzerland

Yariv Yogev

Israel

BaoSheng Yu

China

Demin Yu

China

Danuta Zapolska-Downar

Poland

Wei Zhang

United States of America

Yi Zhang

United States of America

Eugene Zhen

United States of America

Zhiguang Zhou

China

